# Supplementary material for: Phylogeography of Baryancistrus xanthellus (Siluriformes: Loricariidae), a rheophilic catfish endemic to the Xingu River basin in eastern Amazonia
Source: PLoS One. 2021 Aug 27;16(8):e0256677. doi: 10.1371/journal.pone.0256677 (PMC8396747; doi:10.1371/journal.pone.0256677)
Supplement: Attachment — Submitted filename: rev_plos_Bxant.pdf [file pone.0256677.s001.pdf]

The manuscript addresses the evolution of Baryancistrus xanthellus under a microevolutionary perspective. Using mtDNA and nDNA the authors brings a considerable dataset of DNA sequences and depicts a well marked population structure. Conjectures about historical events that could have led to this contemporary scenario are robust. Additionally, the species is highly commercialized in the aquarium trade and conservation issues of B. xanthellus are properly discussed. Considering that aquarium trade is a global issue, as well as conservation of Amazonian biota, this manuscript achieves an importance that goes beyond its regional relevance.

Consequently, I recommend the manuscript to be published by PLOS provided that the following comments are addressed.

Most of my comments concern minor issues on the manuscript and are found below. I would like, however, to bring attention to an important question. The population structure depicted by the analyses are so strong that could be interpreted as signs of an ongoing speciation processes or that B. xanthellus is a species complex. This is a critical issue and should somehow be addressed in the Discussion. Also, the authors should clearly state if the color morphs are coincident with the haplogroups or not. Introduction (lines 80 to 81) states that this variation are related to population structure. Maybe the authors employed both ecological and genetic population concepts of population on the text, and can lead readers to a misunderstanding.

I also would like to straight that the way some of the analyses were conducted may be improved and bring better results (FST and AMOVA, see comments below).

Introduction

Line 63: “zig-zag sinuous” seem to be redundant to me as zig-zag is already sinuous.

133: use “locality” instead of “site” as employed in the whole manuscript

Materials and Methods

Data preparation

164 - 173: I’m not familiar with Phase algorithms but I know that it offers a number of parameters. It is important that these parameters are given on the text.

Population and phylogeographic analyses

180: Parameters of BAPS6 are also poorly described. I strongly recommend the authors to have a look at other papers to see how methods are explained. Number of replications and K values are fundamental and need to be explained. How did K was estimated? Was it based on the haplotype network results? Did authors use other software to build the output like Distruct or Clumpp? Finally, I never used BAPS and I was unable to install or see its documents and for these reasons I cannot assure what are the parameters the should figure on the text. Though I believe it might be close to Structure package. If this is the case, authors should keep in mind that this is an analysis of attribution that is dependent of K calculation, where K is the number of populations. Different

numbers of K should have been compared in order to find the best population structure given the current dataset. So, its of paramount importance to let the reader know how K was found.
185 - 192: AMOVA was employed in a manner that I do not agree and consequently its results are not worthy to be discussed. In fact, AMOVA is usually used in two ways. The first as an exploratory analysis to help the formulation of hypothesis given a data set. The second is to test and compare different hypotheses, i.e. different scenarios of population structure. In this work, an analysis of attribution is performed by BAPS6 and its result is considered to be the most plausible population structure. Hence, AMOVA should be used to go further on BAPS proposed structure and to show in more detail how genetic variation is partitioned among populations. I also believe that FST was used as an exploratory analysis and its results are not relevant. Since FST is also calculated in AMOVA I believe that this analysis could be removed from the paper.
196: Parameters and priors of EBSP should also be stated.

Results
228: Table 3 caption should state what are one or two asterisks.
265-267: This is not correct. Results for cytb are distinctly different.
276: This whole table of results is unnecessary (as stated above - see comments on the use of FST and AMOVA)
279: AMOVA, and not FST, should have been used to test the hypothesis of existing five population hypotheses (as stated above - see comments on the use of FST and AMOVA)
301: “The lack of genetic substructure ‘in’ nuclear markers…” Is this sentence correctly written?

Discussion
Genetic diversity: Maybe this subsection could be merged with Population Structure and Phylogeography as in the very beginning it refers to the main result of population structure (“…for three of the five groups analyzed…”). This is just a suggestion.
Demographic history
439-441: Results of D and Fs can be interpreted further than only deviation from neutrality. Negative values may be interpreted as population expansion as suggested by the haplotype network star-like shape and endorsed by EBSP results.
